# Supplementary material for: Exploiting long read sequencing to detect azole fungicide resistance mutations in Pyrenophora teres using unique molecular identifiers
Source: Sci Rep. 2024 Mar 15;14:6285. doi: 10.1038/s41598-024-56801-z (PMC10943121; doi:10.1038/s41598-024-56801-z)

Supplementary Figure and Tables

Supplementary Table S1. Cq values from quantitative PCR validation of UMI PCR input DNA for sequencing of *Cyp51A* mock community. Positive controls consisted of genomic DNA from an isolate known to contain the mutation being tested. Negative controls consisted of genomic DNA from an isolate known to not contain the mutation being tested. *Cyp51A1* mock community samples consisted of two technical replicates. Error values are standard error of the mean.

Assay	Positive control	Negative control	*Cyp51A* mock community
PtTi-1	22.71	none	14.395±0.085
PtTi-2	20.45	none	12.285±0.075
PtTi-3	22.5	none	13.51±0.11
PtTi-4	23.2	none	14.62±0.15
Pt-Ti-5	23.65	none	14.43±0.045
CYP51A1_F-L489-1	26.26	none	16.16±0.22
CYP51A1_F-L489-3	24.45	none	15.41±0.01

Supplementary Table S2. Cq values from quantitative PCR validation of UMI PCR input DNA for sequencing of five field samples. Positive controls consisted of genomic DNA from an isolate known to contain the mutation being tested. Negative controls consisted of genomic DNA from an isolate known to not contain the mutation being tested. Field samples consisted of two technical replicates. Error values are standard error of the mean.

Assay	Positive control	Negative control	2020-229-1	2020-230-7	2020-231-9	2020-234-3	2020-283-3
Ptt_R1ID1	21.84	none	22.4±0.11	21.58±0.07	23.29±0.05	22.84±0.14	23.18±0.04
Ptm_r5ID12	25.25	none	none	none	none	none	none
CYP51_F489	22.82	none	24.59±0.03	23.85±0.31	24.74±0.20	24.59±0.06	24.28±0.13
CYP51A1_F-L489-1	24.51	none	none	none	none	none	none
CYP51A1_F-L489-3	26.61	none	26.74±3.09	26.37±0.09	26.86±0.10	27.00±0.07	26.60±0.11

Supplementary Figure S1. Nucleic acid alignment of clusters generated from UMI PCR of *Cyp51A* genes from seven isolates of *P. teres* compared to reference sequences when inputting 1 000 000 reads. GSP_F and GSP_R = gene specific primers used for UMI PCR (Table 2). Shading is as follows: black = 100% similar, dark grey = 80-99% similar, medium grey = 60-79% similar, light grey = less than 60% similar. Image created using Geneious 2022.1 created by Biomatters.

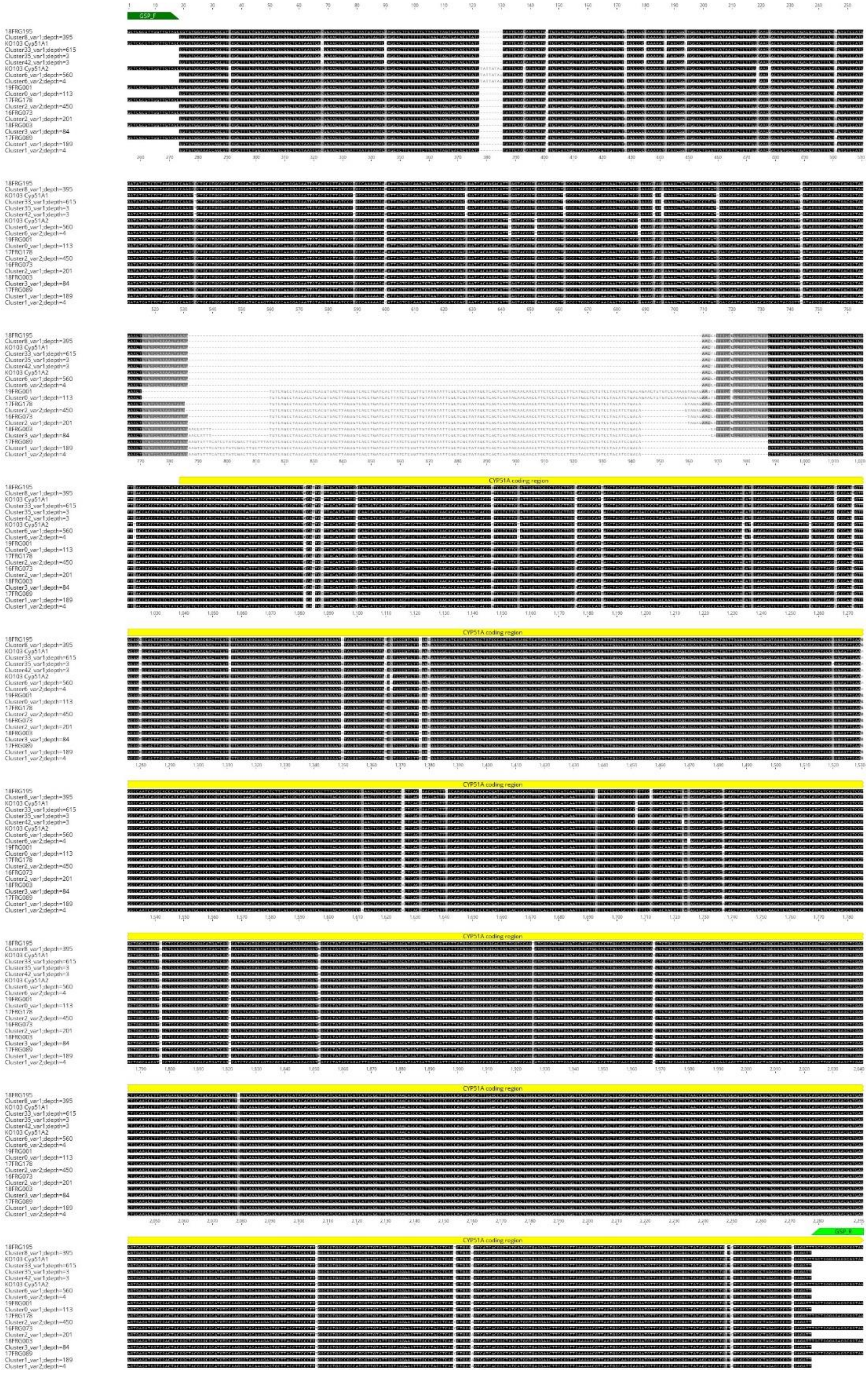
GSP_F
18FRG195
Cluster8_var1;depth=395
K0103 Cyp51A1
Cluster33_var1;depth=615
Cluster35_var1;depth=3
Cluster42_var1;depth=3
K0103 Cyp51A2
Cluster6_var1;depth=560
Cluster6_var2;depth=4
19FRG001
Cluster0_var1;depth=113
17FRG178
Cluster2_var2;depth=450
16FRG073
Cluster2_var1;depth=201
18FRG003
Cluster3_var1;depth=84
17FRG089
Cluster1_var1;depth=189
Cluster1_var2;depth=4
CYP51A coding region
GSP_R

Supplementary Figure S2. Nucleic acid alignment of clusters generated from UMI PCR of *Cyp51A* genes from five leaf samples infected with *Pyrenophora teres* f. *teres* when inputting all reads. Shading is as follows: black = 100% similar, dark grey = 80-99% similar, medium grey = 60-79% similar, light grey = less than 60% similar. Image created using Geneious 2022.1 created by Biomatters.

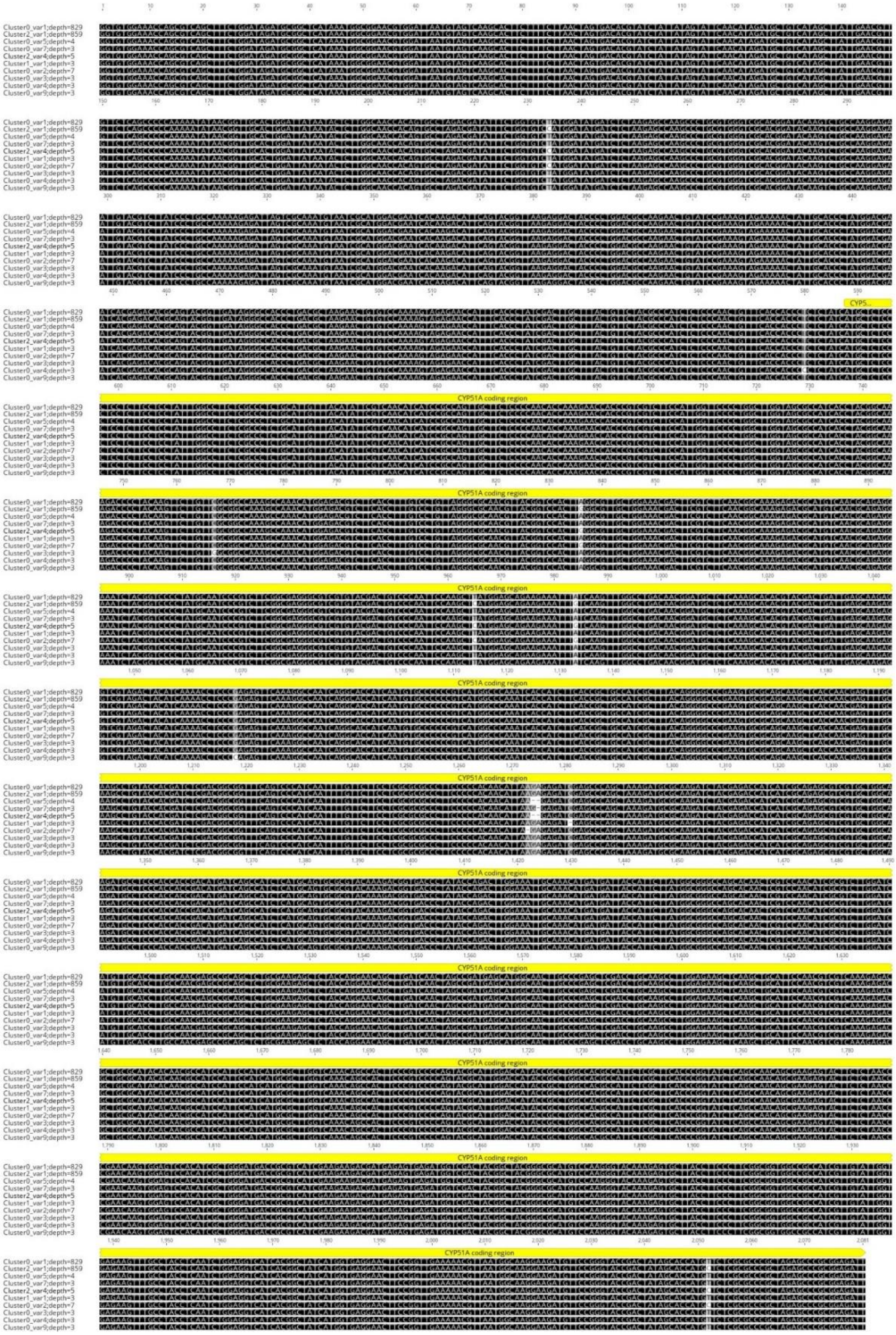
Cluster0_var1;depth=829
Cluster2_var1;depth=859
Cluster0_var5;depth=4
Cluster0_var7;depth=3
Cluster2_var4;depth=5
Cluster1_var1;depth=3
Cluster0_var2;depth=7
Cluster0_var3;depth=3
Cluster0_var4;depth=3
Cluster0_var9;depth=3
CYP5...
CYP51A coding region

Supplementary Figure S3. Sequence length vs. quality scatter plots of input sequences to A) Cyp51A mock community and B) field samples. Figures generated using NanoPlot (https://github.com/wdecoster/NanoPlot).

A

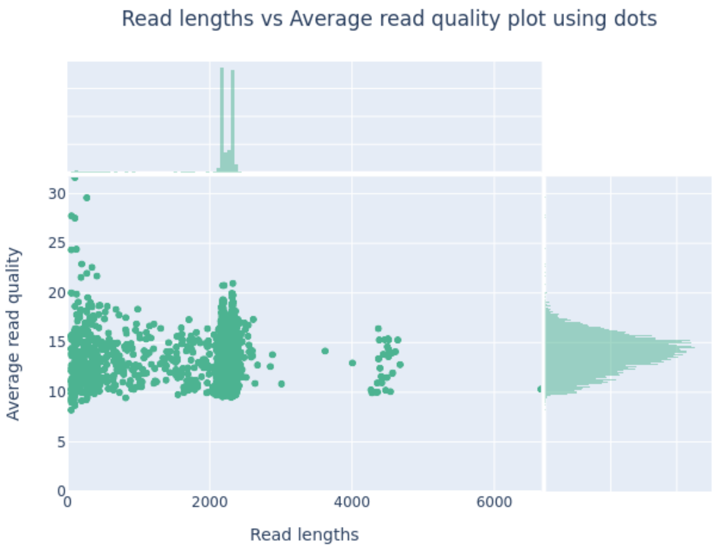

B

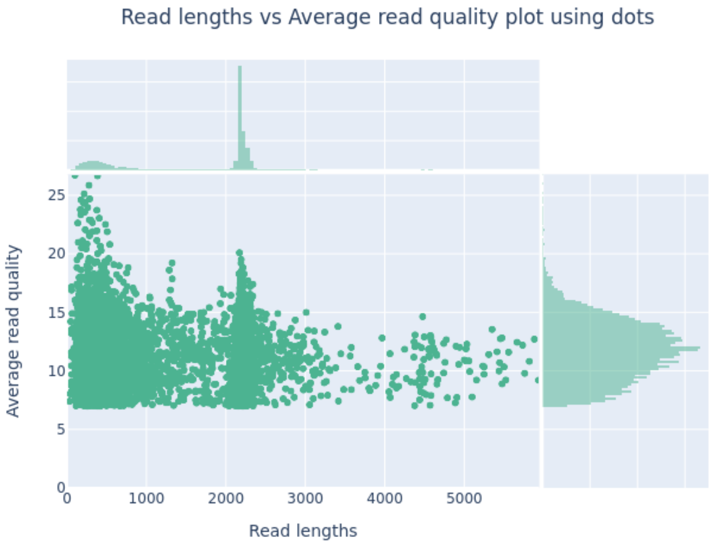

Supplement: Supplementary file 1 — Supplementary Information. [file 41598_2024_56801_MOESM1_ESM.pdf]
